# Charcoal rot in sesame: infection biology, host resistance mechanism, and genomic-enabled strategies for durable resistance breeding

**DOI:** 10.1007/s00122-026-05241-6

**Published:** 2026-04-27

**Authors:** Amjad Ali, Muhammad Azhar Nadeem, Fatih Ölmez, Cemal Kurt, Faheem Shehzad Baloch

**Affiliations:** 1 Department of Plant Protection, Faculty of Agricultural Sciences and Technologies, Sivas University of Science and Technology, Sivas, 58140 Turkey; 2 Department of Biotechnology, Faculty of Science, Mersin University, Yenişehir, Mersin 33343 Turkey; 3 Department of Field Crops, Faculty of Agriculture, Çukurova University, Adana, 01330 Turkey; 4 Department of Genetics, Institute of Biochemistry, Sh. Rashidov Samarkand State University, 140104 Samarkand, Uzbekistan

## Abstract

Sesame (*Sesamum indicum* L.) is a globally important oilseed crop, but its production is constrained by charcoal rot, caused by the soil-borne fungus *Macrophomina phaseolina.* The pathogen’s exceptionally broad host range, long-term soil persistence through microsclerotia, and increased aggressiveness under high temperature and drought make charcoal rot a major destructive constraint in sesame-growing regions. This review integrates current knowledge on the biology, epidemiology, infection processes, and genetic population variability of *Macrophomina* spp. in relation to charcoal rot development in sesame. We also summarize key host resistance mechanism including pathogen perception, cell wall reinforcement, phenylpropanoid-mediated defense, antioxidant responses, and associated physiological and molecular adaptations. Particular attention is given to the challenges of resistance screening under variable environmental conditions, including heat- and drought-associated disease expression and pathogen diversity, which complicate the identification of stable resistance sources. The review further examines progress in sesame improvement through germplasm characterization, mutation breeding, interspecific introgression, high-throughput phenotyping, and genomic-assisted approaches such as QTL mapping, genome-wide association studies (GWAS), marker-assisted selection, genomic selection, and functional validation. Integrating these tools with multi-omics and gene-editing strategies offers a promising route for accelerating the development of durable, climate-resilient charcoal rot resistance cultivars. Broader use of diverse germplasm, standardized multi-environment phenotyping, and international collaboration will be essential for sustainable resistance breeding and future sesame production.

## Introduction

Sesame is one of the oldest cultivated oilseed crops, valued for its high oil content, nutritional quality, and economic importance. Its seeds contain 37–63% oil contents (depends on cultivar type), rich in antioxidants like sesamin and sesamol, making it highly desirable for human consumption and industrial applications (Wei et al. 2022; Angamuthu et al. 2025). Sesame oil is widely used in cooking, cosmetics, and pharmaceuticals, contributing to its global demand (Kim et al. 2024; Sanni et al. 2026; Pakbaz et al. 2026). It is primarily cultivated in tropical and subtropical regions and a key cash crop for smallholder farmers in countries such as India, Sudan, and Nigeria. Strong market demand for sesame seed and oil has increased its economic value and inforced its importance in global oilseed markets (Ölmez and Sevilmiş 2021). Previous studies report that sesame seeds contain approximately 21.9% protein and 61.7% oil, and are also a good source of minerals, particularly iron (Fe) and calcium (Ca) (Wei et al. 2022).

Nutritionally, sesame oil is dominated by unsaturated fatty acids: per 100 g oil, ~ 14.2 g saturated, ~ 39.7 g monounsaturated, and ~ 41.7 g polyunsaturated fat, indicating that ~ 81–82% of total fat is unsaturated (https://foodstruct.com/foodinfographic/en_sesame-oil.jpg.webp/). The lipid profile is mainly oleic (≈36–42%) and linoleic (≈42–48%) acids, with lower levels of palmitic (≈8–12%) and stearic (≈5–6%) acids (Bakal 2022). In addition to its favorable fatty acid composition, sesame contains bioactive lignans and antioxidant constituents that support its recognition as a nutritionally valuable crop. Economically, sesame production and trade have expanded due to strong demand in food and processing industries. Food and agriculture organization statistics (FAO 2024) indicate that global sesame seed production reached ~ 6.74 million tonnes in 2022 (6.67 million tonnes in 2021), led by major producers including Sudan, India, and Myanmar. International demand is reflected in trade data: Global trade in sesame seed (HS 120740) was about USD 4.36 billion in 2023, with top exporters including India (≈USD 489 M), Nigeria (≈USD 459 M), and Pakistan (≈USD 414 M) (https://oec.world/en/profile/hs/sesamum-seeds?utm/). In Europe, consumption and import demand are concentrated in markets such as Germany and Greece, with the Netherlands serving as a key import and re-export hub, and there is a growing premium segment for certified and organic sesame (https://www.cbi.eu/market-information/grains-pulses-oilseeds/sesame-seeds/market-potential/).

However, productivity remains low due to susceptibility to diseases and abiotic stresses. Improving its resilience to biotic stresses, particularly charcoal rot, is essential to increase yield and ensure sustainable production. Charcoal rot, caused by *M. phaseolina*, is one of the most destructive disease of sesame across major growing areas. The pathogen invades the vascular system, leading to vascular blockage and tissue necrosis, and can cause 5 to 100% yield loss under severe conditions (Prasad et al. 2022; Dolatkhah and Ahmadpour 2024). The pathogen has been reported to cause in yield loss upto 50% in Tamilnadu and 42–45% in India (Vinothini et al. 2020). The pathogen is reported in sesame crop from the several countries of the globe including Australia, Brazil, Bangladesh, Colombia, China, Cyprus, Cuba, Egypt, Ecuador, Greece, Ethiopia, Honduras, Iran, India, Israel, Iraq, Kenya, Japan, Myanmar, Mexico, Nigeria, Nicaragua, Paraguay, Pakistan, Republic of Korea, Sudan, Sri Lanka, Tanzania, Syria, Türkiye, Thailand, USA, Uganda, and Venezuela (Bashir 2018; Langham et al. 2021; Adorada et al. 2025). The biotic stress thrives in high temperatures and water-limited conditions, impacting seed weight, oil content, and marketability. Traditional breeding approaches (phenotypic selection and hybridization) have contributed to improvement in yield and stress tolerance; however, their effectiveness for durable resistance breeding is often constrained by several limitations. The key drawbacks include: restricted genetic variation in elite germplasm and crossing barriers with wild relatives, quantitative and polygenic inheritance of many resistance and yield-related traits often with small-effect loci, and strong genotype × environment interactions and low-to-moderate heritability under variable field disease pressure, which reduce selection accuracy, and moreover, the late, inconsistent, or costly phenotyping for many diseases/stresses and frequent confounding by escape mechanisms; linkage drag and unfavorable correlations when introgressing resistance from diverse sources, leading to penalties in yield or quality; and the long breeding cycle, particularly when pyramiding multiple resistance genes or combining resistance with high agronomic performance. Despite these constraints, combining ability analysis and heritability estimates remains valuable for identifying parents and cross combinations with superior potential, highlighting traits such as plant height, seed yield, and oil content (Mahmoud et al. 2024; Nanda et al. 2025).

Modern tools, such as molecular markers, have revolutionized breeding by enabling the identification of resistance-linked traits. Techniques like RAPD, AFLP, SSRs, and SNPs have been instrumental in genetic diversity studies and marker-assisted selection (MAS), which accelerates the development of resistant varieties (Bhat et al. 1999; Parsaeian et al. 2011). GWAS application further enhances breeding by uncovering genetic loci associated with complex traits, including disease resistance, oil quality, and yield. Studies using GWAS have identified significant SNPs and candidate genes linked to traits like seed coat color and stress tolerance, offering precise molecular targets for breeding programs (Guden et al. 2022; Rauf et al. 2024; Elsafy et al. 2025). Integrating conventional and modern techniques, such as GWAS and MAS, with biotechnological advancements like gene editing, provides new opportunities to combat charcoal rot.

Several reviews have summarized *M. phaseolina* biology, epidemiology, host interaction, and management across crops, providing a valuable foundation for understanding charcoal rot as a broad pathosystem (Radadiya et al. 2021). In parallel, recent reviews have discussed progress in sesame improvement and the expanding genomics toolbox available for trait discovery and breeding (Marquez et al. 2021). However, a sesame-focused synthesis that explicitly connects (i) *M. phaseolina* population variability and infection biology, (ii) sesame resistance mechanisms and phenotyping constraints under heat–drought stress, and (iii) genomic-enabled breeding routes (QTL/GWAS/GS and functional validation) remains limited; moreover, fast-growing transcriptomic and genomic evidence on sesame–Macrophomina interactions has not been consistently consolidated into actionable priorities for resistance deployment (Rauf et al. 2024). Therefore, this review compiles and integrates current evidence on charcoal rot in sesame and proposes a coherent framework from pathogen biology and resistance mechanisms to marker-assisted and genome-informed breeding strategies to support development of durable, climate-resilient resistant cultivars.

## Review methodology

This article is a narrative review that synthesizes published evidence on charcoal rot in sesame and related oilseed pathosystems. Relevant studies were selected to cover (i) pathogen taxonomy, infection biology, disease cycle, and epidemiology; (ii) host responses associated with resistance or tolerance; and (iii) genetic improvement strategies, including phenotyping approaches and genomic-enabled breeding tools. Priority was given to primary studies that clearly described plant materials, pathogen isolates, experimental conditions, and phenotyping or molecular methods, and that reported quantitative outcomes (e.g., disease incidence or severity, plant survival, yield effects, or marker–trait associations). Evidence was extracted, grouped by theme, and synthesized qualitatively to highlight areas of consensus, methodological limitations, and key research gaps.

Cultivar reactions summarized in Table 1 were recorded as reported by the original sources under their stated screening conditions. Field photographs illustrate typical charcoal rot symptoms observed in sesame fields in Diyarbakir (Türkiye) and were used for visual documentation only.

**Table 1:** Sesame cultivars/varieties reported as resistant or tolerant to charcoal rot (*Macrophomina* spp.) in screening or breeding studies

Country/region	Cultivar / variety	Breeding pathway (as reported)	Reported reaction to charcoal rot	References
Pakistan	TS-5	Conventional (selection; released cultivar)	Resistant	Anwar et al. (2012)
Iran	Behbahan	Conventional (cultivar recommendation for infested areas)	Suggested as preferable/superior under infestation	Moslemi et al. (2023)
Iran	Dashtestan	Conventional (cultivar recommendation for infested areas)	Suggested as preferable/superior under infestation	Moslemi et al. (2023)
India	RT-46	Conventional (field/varietal screening)	Tolerant	Gupta et al. (2018)
India	RT-0125	Conventional (field/varietal screening)	Tolerant	Gupta et al. (2018)
India	MT-75	Conventional (field/varietal screening)	Tolerant	Gupta et al. (2018)
India	TKG-22	Conventional (field/varietal screening)	Tolerant	Gupta et al. (2018)
India	Nirmala	Conventional (field/varietal screening)	Tolerant	Gupta et al. (2018)
India	GT-10	Conventional cultivar (used as resistant check/donor in molecular work)	Classified as resistant	Radadiya et al. (2021)
China	Zhongzhi No. 13	Conventional cultivar (used as resistant parent for MAS/QTL work)	Reported high resistance; used as resistant parent in QTL mapping	Wang et al. (2017)

## Pathogen biology and epidemiology of *M. phaseolina*

### Taxonomy, morphology, and key biological features

*M. phaseolina* (family, *Botryosphaeriaceae*) is recognized as a globally important pathogen, and current evidence does not support subdivision into subspecies or physiological races based on morphology or genomic characterization (Marquez et al. 2021). Recent phylogenetic work has also recognized additional Macrophomina species *M. pseudophaseolina* and *M. euphorbiicola* reported from multiple hosts, e.g., okra/groundnut/cotton/castor and other crops (Pennerman et al. 2025; Alizadeh et al. 2025). Morphologically, *M. phaseolina* produces hyaline to light/dark brown septate hyphae with branching typically at right angles and a constricted branch origin, and it forms abundant microsclerotia (spherical to oblong; light brown when young, darkening to brown/black with age) (Lakhran et al. 2018). Pycnidia are reported as comparatively rare in nature and described as dark, rough, globose/irregular, often beaked and isolated; isolates can vary in colony traits on media, but ITS amplification has supported assignment to a single species (Almomani et al. 2013). The key biological features emphasized include its role as a globally distributed, generalist soil-borne pathogen with an exceptionally broad host range (≥ 500 plant species across > 100 families) and major impacts under high temperature (30–35 °C) and low soil moisture (Ghosh et al. 2018; Papini et al. 2025). The fungus persists primarily as microsclerotia, which function as the main inoculum and can survive in soil for up to 15 years, enabling long-term carryover (Shirai and Eulgem 2023). After infecting roots (often at the seedling stage), it colonizes and disrupts the vascular system, impairing water/nutrient transport and leading to characteristic field symptoms such as leaf yellowing/senescence (often remaining attached), cortical tissue sloughing near the lower stem/taproot, gray discoloration from microsclerotia abundance, and potentially premature plant death (Short et al. 1978; Bristow and Wyllie 1986; Collins et al. 1991). Genetically, despite the reported absence of sexual reproduction, populations show high diversity, potentially supported by parasexual processes (hyphal fusion/heterokaryon formation) that contribute to observed variability.

### Infection process, disease cycle, and survival structures

**Fig. 1:**
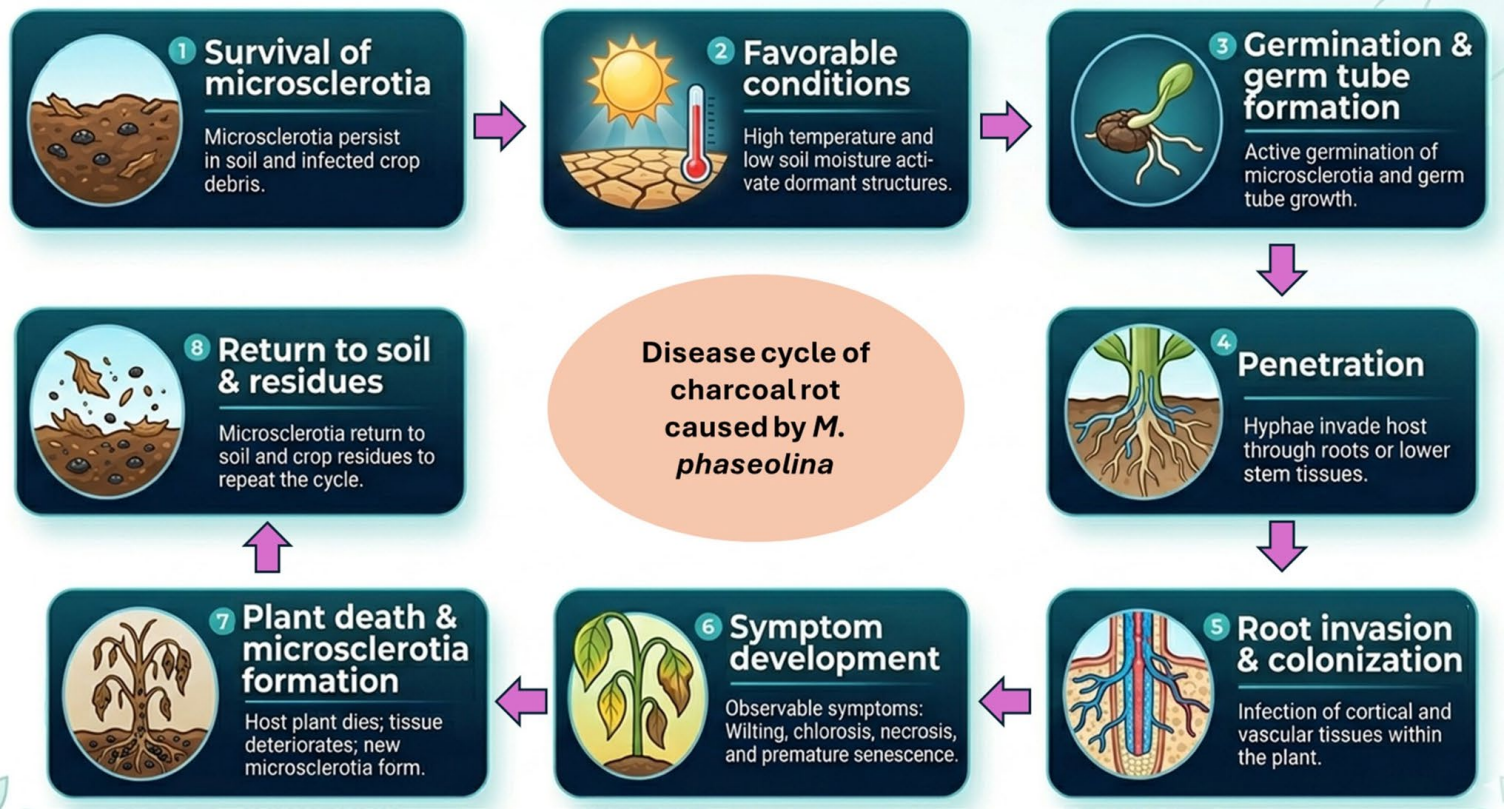
Stepwise disease cycle of charcoal rot caused by *M. phaseolina* in sesame. The diagram illustrates the major stages of disease development, including (1) survival of microsclerotia in soil and infected crop debris, (2) favorable environmental conditions, especially high temperature and low soil moisture, (3) germination of microsclerotia and germ tube formation, (4) penetration through roots or wounded basal tissues, (5) colonization of root and lower stem tissues, (6) symptom development including wilting, chlorosis, necrosis, and premature senescence, (7) plant death and formation of new microsclerotia in infected tissues, and (8) return of inoculum to soil and crop residues, enabling persistence between cropping seasons

*M. phaseolina* enters in host plants through natural openings or wounds, colonizes tissues by producing cell wall-degrading enzymes, and produces toxins (Islam et al. 2012; Ghosh et al. 2018). As infection progresses, the vascular system becomes blocked, leading to symptoms like leaf yellowing and wilting. The pathogen forms new microsclerotia within decayed plant tissues, which are released back into the soil and spread to new hosts. Microsclerotia then enter a dormant state under unfavorable conditions, making the pathogen highly persistent between cropping seasons and destructive, especially under favorable conditions for decrease development (Lodha and Mawar 2020). Figure 1 illustrates the sequential stages of the charcoal rot disease cycle in sesame, from microsclerotial survival in soil and crop debris to root penetration, tissue colonization, symptom development, and return of inoculum to the soil. Short et al. (1980) reported that *M. phaseolina* is capable of persisting on plant residues located at or just beneath the soil surface for a period approaching two years. However, their investigation did not explore whether the pathogen retained its infective potential after this extended survival period. Complementing these findings, research carried out in Brazil by Reis et al. (2014) demonstrated that the microsclerotia of *M. phaseolina* could endure on infected crop debris for nearly 35 months. Despite this prolonged survival, the ability of the pathogen to cause disease notably declined after about six months. These observations suggest that while the fungus can persist in the environment for extended durations, its virulence may diminish over time, potentially influencing disease epidemiology and management strategies.

The sexual reproductive stage of *M. phaseolina* has not been reported to date. Occasionally, pycnidia the asexual fruiting bodies have been reported on various host plants (Dhingra and Sinclair 1978), as well as under laboratory culture conditions using artificial media such as potato dextrose agar (Gaetán et al. 2006) and water-agar-leaf agar media (Chidambaram and Mathur 1975; Ma et al. 2010). However, the precise contribution of pycnidia to the pathogen’s disease development and life cycle is still unclear and requires further investigation. The conidia produced by *M. phaseolina* are typically transparent (hyaline) and exhibit an ellipsoid to ovoid shape, measuring approximately 14 to 30 µm in length and 5 to 10 µm in width (Crous et al. 2006). Experimental studies have demonstrated that these conidia are capable of infecting soybean seedlings under controlled laboratory conditions (Ma et al. 2010), although their exact role and significance in the natural disease progression (including in sesame) have not been definitively established.

### Environmental and agronomic factors influencing disease severity

The progression and intensity of charcoal rot are significantly affected by environmental factors. Elevated soil temperatures (exceeding 28 °C) and reduced soil moisture are the primary factors facilitating the proliferation of *M. phaseolina* (Gupta et al. 2012; Pedroncelli et al. 2024). Drought stress compromises host plants, increasing their vulnerability to infection. Poorly drained soils, elevated salinity, and diminished organic matter content also exacerbate disease prevalence (Ranjan et al. 2024). Crop residue management is critical, as contaminated material remaining in the field serves as a reservoir for microsclerotia. Moreover, the persistent cultivation of susceptible hosts increases inoculum density in the soil. Climate change-induced fluctuations in temperature and precipitation are anticipated to intensify the incidence of charcoal rot in sesame cultivation areas, presenting a considerable challenge for disease control (Pandey and Basandrai 2021). Beyond their direct effects on disease severity, high temperature, drought, and cropping system variables may also act as selective forces shaping local *M. phaseolina* populations, which helps explain why regional isolate collections can differ in aggressiveness and adaptation (Sexton et al. 2016; Ortiz et al. 2023; Farag et al. 2025).

### Economic impact of charcoal rot on sesame production

**Fig. 2:**
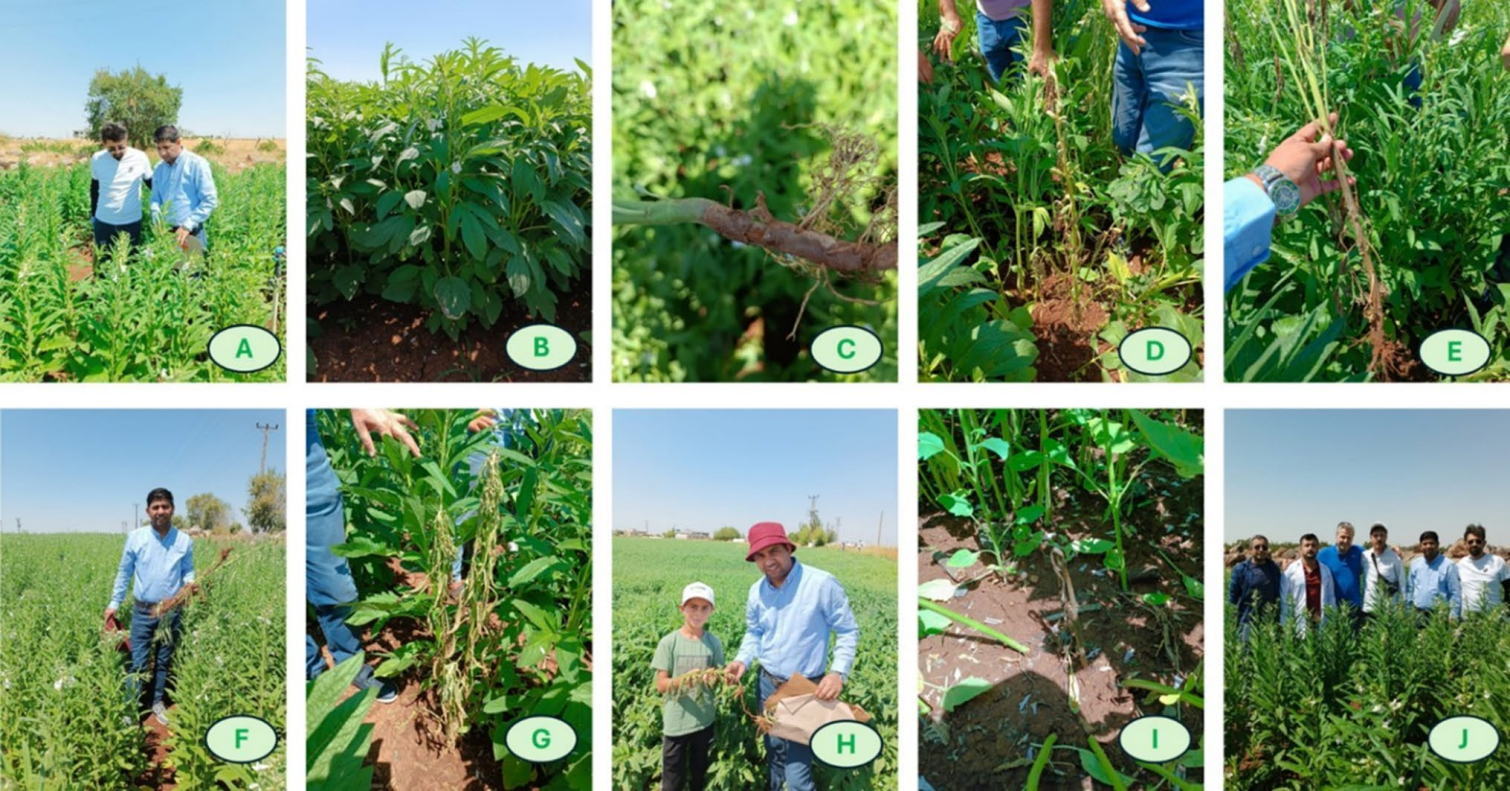
Field documentation of charcoal rot symptoms in sesame at different growth stages in Diyarbakır, Türkiye. (A) Field survey and in situ inspection of sesame plants in a naturally infected field. (B) Healthy sesame plant showing normal canopy development and green foliage, used as a visual reference. (C) Early stem and root symptom expression, with brown necrotic discoloration developing on the lower stem/taproot region. (D) Diseased plant at the flowering to early capsule-setting stage showing reduced vigor, lower stem discoloration, and early wilting. (E) Uprooted infected plant showing root deterioration and brown to dark discoloration extending from the root system to the basal stem. (F) Diseased plant in the field with visible wilting and loss of canopy turgor during the reproductive stage. (G) Advanced symptom expression at capsule development to maturity, characterized by severe wilting, leaf drying/senescence, and near-complete collapse of the aboveground plant parts. (H) Collection of symptomatic plants during field assessment. (I) Basal stem lesion and lower stem/root discoloration near the soil line, a diagnostic feature supporting field identification of charcoal rot. (J) Field survey team during disease assessment. Visible symptoms in this figure span the reproductive period, especially from flowering through capsule development and maturity, when charcoal rot symptoms become most evident under field conditions

Charcoal rot imposes substantial economic losses in sesame through both yield and quality penalties. Reported yield losses range from 10% under moderate disease pressure to total crop failure in severe outbreak driven by premature plant senescence and reduced capsule filling (Yan et al. 2021). Infected plants produce fewer and smaller seeds, while oil content declines and free fatty acid levels increase, reducing both oil recovery and sale price at farm gate and processing stages. The symptoms observed during our recent field survey in Diyarbakir, Türkiye, are shown in Fig. 2. Recent surveys also highlight the need to consider *Macrophomina* species diversity: *M. phaseolina* remains dominant in Iran (Moslemi et al. 2024). The pathogen infects at any growth stage, secreting enzymes and toxins that disrupt plant tissues, leading to significant yield loss. Kundu et al. (2025) reported an emerging charcoal rot pathogen in sesame, identifying *M. tecta* as the causal agent based on symptomatic outbreaks observed in eastern India during 2023–2024 and subsequent isolation of 18 fungal isolates from diseased plants. Using an integrative workflow morphology/ultrastructure, pathogenicity (Koch’s postulates), and multilocus sequencing/phylogeny, they showed that isolates from infected sesame clustered in a distinct phylogenetic clade with *M. tecta* using five loci (ITS, tef1-α, act, cmd, tub2), constituting the first global report of *M. tecta* infecting sesame and expanding its known host range beyond cereals and legumes.

Reported loss ratios vary widely among studies and should be interpreted with caution because they are strongly influenced by host genotype, environment, and screening methodology. Recent breeding work in sesame showed significant differences in infection percentage among parental lines and hybrids, indicating that cultivar background can substantially alter the degree of damage expressed under pathogen pressure (Mahmoud et al. 2024). In addition, disease development is intensified by low soil moisture and heat stress, conditions that favor *Macrophomina* infection and accelerate symptom expression, thereby increasing yield penalties in susceptible genotypes (Pedroncelli et al. 2024; Farag et al. 2025). Methodological differences also contribute to variation: Greenhouse pathogenicity assays and artificial inoculation often impose more uniform and severe disease pressure than natural field infection, whereas field estimates are further modified by sowing time, crop growth stage at infection, and disease escape (Aydoğdu and Kurbetli 2024). Therefore, the wide range of reported losses reflects genotype x environment x method interactions rather than a single fixed damage level, underscoring the need for standardized multi-environment phenotyping when comparing the harm caused by charcoal rot.

Beyond direct revenue losses, charcoal rot increases production costs (e.g., re-sowing, irrigation, fungicides/seed treatments, and soil amendments strategies) (Ijaz et al. 2013; Lodha and Mawar 2020). Quality downgrades can trigger price discounts or rejection in export markets when seeds fail to meet international standards, while long-lived microsclerotia reduce land-use efficiency by necessitating extended rotations or fallow. To mitigate these economic impacts, management should prioritize prevention and inoculum reduction through an integrated strategy: deployment of tolerant/resistant cultivars where available (e.g., Behbahan and Dashtestan in affected areas of Iran; Moslemi et al. 2024), use of disease-free seed and appropriate seed treatments, rotation with non-host crops and residue sanitation, and agronomic practices that alleviate heat–drought stress (timely irrigation, balanced nutrition, and organic amendments to improve soil health) (Vashisht et al. 2023; Pandey et al. 2023). Biological control agents such as *Trichoderma* spp., *Bacillus subtilis*, and *Pseudomonas fluorescens* can further suppress *M. phaseolina* and reduce reliance on chemical inputs, improving the cost-effectiveness of control in resource-limited systems (Karibasappa et al. 2020; Nanda et al. 2024).

### Symptoms and diagnostic features in sesame

Infection can occur from seedling emerging through vegetative growth; however, visible symptoms are most commonly expressed during the reproductive phase, particularly from flowering through capsule development and ripening. Early indications include leaf chlorosis, reduced vigor, brown to reddish discoloration of roots and the lower stem, often accompanied by wilting (Gupta and Chauhan 2005; Short et al. 1978). As the disease progresses, foliar symptoms become more pronounced including premature senescence, severe wilting, and early plant death. These symptoms can resemble other biotic and abiotic stresses (e.g., drought stress or nematode damage); diagnosis should be supported by stem/root examination and the presence of abundant microsclerotia. A useful field distinction is that leaves frequently remain attached to the petioles after plant death (Mengistu 2015). Representative field symptoms documented during our Diyarbakır (Türkiye) survey are shown in Fig. 2, with panels C-I illustrating symptom progression from early lower stem/root discoloration and wilting at flowering to severe senescence, plant collapse, and basal stem necrosis during capsule development and maturity.

The term "charcoal rot" is derived from the characteristic gray to silvery appearance of lower stems and roots which results from dense microsclerotia formation on and within affected tissues (Gupta et al. 2012). In field conditions, symptoms are typically more prevalent in areas subjected to environmental stress, such as field edges, compacted soils, and sloped areas. Moreover, disease severity generally increases under elevated temperatures combined with low soil moisture (Meyer et al. 1974; Mihail 1989). Plant maturity can also influence symptoms expression; early-maturing plants that flower and fill capsules during relatively cooler periods may experience reduced stress and consequently exhibit less severe charcoal rot and yield loss (Mihail 1989; Smith and Carvil 1997). Historically, thin black zone lines in the stem cortex were considered a diagnostic symptom; however, these zone lines are now known to be associated with *Diaporthe longicolla* rather than charcoal rot disease, improving the accuracy of disease diagnosis (Olsen et al. 2015).

### Molecular approaches for early detection and identification

Accurate and early detection of *M. phaseolina* is crucial for disease management. PCR and hybridization methods using species-specific oligonucleotide primers enable rapid identification (Babu et al. 2007). Recently, primers have been developed to differentiate species within the *Macrophomina* genus, including *M. phaseolina* (Santos et al. 2020). Real-time qPCR assays using SYBR Green and TaqMan have been designed for pathogen quantification in both rhizosphere soil and host tissues (Burkhardt et al. 2018). These tools allow direct estimation of *M. phaseolina* populations from soil DNA without culturing (Marquez et al. 2021). Beyond disease diagnosis, molecular detection tools can directly strengthen breeding pipelines by confirming pathogen identity, distinguishing genetically diverse isolate groups, and helping breeders assemble representative inoculum panels for reliable resistance screening. In sesame, species-specific marker-based characterization has already been proposed as a useful bridge between pathogen diagnostics and breeding for charcoal rot resistance, because accurate isolate identification improves both screening precision and interpretation of resistance performance across environments (Ali et al. 2025b, c, a; Pennerman et al. 2025). A recent sesame-focused study from Türkiye also validated species-specific PCR detection, with all seven isolates collected from symptomatic plants in Diyarbakır and Şanlıurfa yielding the expected 350-bp amplicon, supporting rapid diagnosis in breeding and epidemiological surveys (Ali et al. 2025b, c, a).

### Genetic diversity and population structure of *Macrophomina* spp. and *M. phaseolina*

Despite being traditionally regarded as predominantly asexual, *M. phaseolina* exhibits substantial genetic and phenotypic diversity across its global range. Early population studies using molecular markers, such as RFLP, RAPD, AFLP, documented high polymorphism among isolates, while later multilocus sequence analyses and genome-wide approaches have provided stronger resolution of lineages, gene flow, and potential adaptive structure (Khan et al. 2017). This diversity is likely driven by parasexualism via hyphal fusion (Almeida et al. 2001). Phylogenetic analyses using rDNA ITS sequences demonstrate significant evolutionary divergence among global isolates. While some studies link genetic variation to geographic origin (Mahdizadeh et al. 2012), others report no correlation with morphology, host, or location (Marquez et al. 2021). A major advance in the global understanding of Macrophomina diversity came from a multilocus analysis (ITS, TEF1-α, ACT, CAL, TUB) of 189 isolates collected from 23 hosts and 15 countries, which resolved two well-defined clades: *M. phaseolina* sensu stricto and a second lineage described as *M. pseudophaseolina* (Sarr et al. 2014). Notably, that study reported no consistent association between genotype and host or geographic origin and demonstrated that distinct *Macrophomina* lineages can occur on the same host at the same location. Furthermore, Reznikov et al. (2019) reported that Brazilian *M. phaseolina* isolates show high haplotype diversity with multiple genotypes sometimes detected within a single infected root system. This heterogeneity in virulence and enzyme secretion supports using genotype-informed, multi-isolate assays for reliable resistance screening.

Recent studies further indicate that pathogen population differentiation should be interpreted not only in terms of geography or host origin, but also in relation to local environmental and agronomic selection pressures. A population-genomic study across the USA, Puerto Rico, and Colombia identified five genetic groups of *M. phaseolina* and showed that climatic diversification contributed to genetic structure beyond host association alone (Ortiz et al. 2023). Likewise, regional comparisons have shown that isolates from contrasting production zones differ in temperature response and pathogenic behavior; for example, southern US isolates displayed greater growth at 40 °C than northern isolates, supporting the view that thermal adaptation contributes to regional population structure (Sexton et al. 2016). Agronomic context may further reinforce this differentiation, because long-term tillage and soil management systems can alter soil biological properties and the incidence of charcoal rot, thereby modifying the selective environment in which *M. phaseolina* persists (Perez-Brandan et al. 2012). Collectively, these findings suggest that climatic conditions, especially heat and drought, and farming systems should be considered alongside host and geographic origin when interpreting population variability and designing region-specific resistance screening strategies.

Genomics-based comparisons further suggest that *M. phaseolina* may not be uniformly “generalist” in practice, with some genotypes showing host preference and lineage-specific gene content. Comparative genome assemblies and resequencing across hosts identified substantial genomic variation and candidate genes associated with a strawberry-preferential genotype, supporting the hypothesis that host-associated differentiation can occur within *M. phaseolina* populations (Burkhardt et al. 2019). In parallel, broader genus-level population genomics has been used to refine species boundaries, develop new diagnostic markers, and infer recombination-related signals (reported as evidence consistent with meiotic recombination) and cryptic specialization among *Macrophomina* lineages (Pennerman et al. 2025). Collectively, these findings indicate that (i) “*M. phaseolina*” in the field may represent a complex of lineages/species, (ii) population structure may be subtle and scale-dependent, and (iii) virulence variability is sufficiently large that resistance screening and management recommendations should be informed by locally representative isolate panels rather than single-isolate testing. Importantly, this creates a critical contrast with earlier multilocus studies that found no consistent association between genotype and host or geography (Sarr et al. 2014), suggesting that *M. phaseolina* behaves as a broad host range pathogen at coarse taxonomic scale but may contain genomically distinct lineages with partial host preference and different risks for resistance erosion when examined at higher resolution (Pennerman et al. 2025).

For sesame-focused research and management, future work should prioritize multilocus or genome-wide genotyping to improve identification and resolve local population structure beyond what ITS alone can provide (Sarr et al. 2014). Sampling should be geographically stratified and included isolates from sesame and key rotation crops, with accompanying metadata on temperature regime, rainfall/aridity, soil properties, irrigation practices, and cropping history, to assess host-associated structure, genotype–environment relationships, and gene flow (Burkhardt et al. 2019). Resistance screening should use multi-isolate panels representing dominant local genetic clusters and highly virulent phenotypes, with periodic updating as new lineages are detected (Ortiz et al. 2023). Finally, standardized isolate metadata (GPS, host/cultivar, and soil climate management descriptors) should be recorded to support genotype–environment and genotype–virulence analyses that inform region-specific recommendations.

Importantly, this population variability has direct guiding significance for sesame resistance breeding. Pathogen populations adapted to different agroecological zones may vary in virulence, thermal tolerance, and stress-associated fitness, which can influence both the expression and durability of host resistance. Population-genomic evidence indicates that climatic variables contribute to genetic clustering and diversification in *M. phaseolina* populations (Ortiz et al. 2023), and climate change projections further suggest that warming and aridity may expand environments favorable for aggressive charcoal rot populations (Farag et al. 2025). This lineage and virulence diversity also increases the risk that resistance effective against one isolate or local population may be less effective against others, especially when pathogen populations differ in host preference, aggressiveness, or environmental adaptation. Consequently, apparent resistance breakdown may occur when breeding programs rely on single-isolate screening rather than testing sesame germplasm against genetically diverse, regionally representative pathogen panels. Accordingly, breeding programs should avoid reliance on single-isolate assays and instead use regionally representative, multi-isolate panels when identifying resistant sesame germplasm, validating QTLs, and selecting parents for durable resistance breeding.

### Molecular mechanisms underlying pathogenicity and virulence

Approximately 12% of *M. phaseolina*'s genome encodes genes related to host interactions, including those involved in adhesion, cell wall degradation, signal transduction, and toxin production. ABC transporters protect the pathogen from host-derived compounds while aiding nutrient uptake (Morschhäuser 2010). Environmental stress, such as hyperosmotic conditions, triggers conidia production through MAP kinase MPH_01444 and regulatory genes MPH_03305 and MPH_10325 (Islam et al. 2012; Ghosh et al. 2018). Adhesion is mediated by cellulose-binding elicitor lectins, transglutaminase-like proteins, and hydrophobic class II proteins (Marquez et al. 2021; Sadhana et al. 2024). The pathogen triggers host immunity using PAMPs such as transglutaminase-like proteins with conserved 13-amino acid motifs, prompting responses like phytoalexin synthesis and CWDE inhibition. In turn, *M. phaseolina* utilizes a cAMP-dependent pathway and enzymes like salicylate-1-monooxygenase to counter host defenses (Dahikar and Nagarkar 2024). During host penetration, it synthesizes phytoceramides to stabilize membranes under stress (Chaudhary et al. 2023). Its virulence is largely attributed to an extensive array of hydrolytic enzymes cellulases, peroxidases, laccases which enable effective plant tissue degradation. *M. phaseolina* has the largest known collection of cellulolytic and lignin-degrading enzymes among sequenced fungi, surpassing *Aspergillus nidulans* and *Neurospora crassa* (Marquez et al. 2021). These enzymes are packaged in vesicles for efficient deployment. The pathogen also produces potent toxins like patulin, botryodiplodin, and especially phaseolinone, which mimic fungal disease symptoms (Salvatore et al. 2020; Khambhati et al. 2023). Its adaptability is reinforced by detoxification genes (e.g., superoxide dismutase, cytochrome P450) and resistance genes for antibiotics and oxidative stress (Ghosh et al. 2018; Marquez et al. 2021). Nitric oxide production, mediated by a nitric oxide synthase-like gene, further supports host colonization, as shown in jute (Sarkar et al. 2014). Appressorium-mediated penetration is powered by turgor pressure and supported by CWDEs and toxins (Chaudhary et al. 2023). This process is governed by cAMP and MAPK pathways. MAC1, protein kinases MPH_00397/07566, and PMK1 are essential for surface recognition and invasive growth (Islam et al. 2012; Xu and Hamer 1996; Ghosh et al. 2018). Heterotrimeric G-proteins further modulate these signaling cascades.

Functional genomics and proteomics are critical to identifying novel virulence factors and understanding how *M. phaseolina* overcomes host defenses. Recent transcriptomic studies in cultivated, wild, and hybrid sesame genotypes infected with *M. phaseolina* have identified key resistance genes related to cell wall reinforcement and phenylpropanoid pathways (Najar and Gangophadhay 2024). Structural modeling of sesame R-proteins suggests that amino acid substitutions near conserved NB-ARC motifs alter ligand binding and immune signaling (Dutta et al. 2020), shedding light on mechanisms underlying charcoal rot resistance.

## Current status of sesame improvement and resistance to charcoal rot

Sesame improvement for charcoal rot resistance remains largely dependent on conventional selection supported by multi-environment screening, as resistance expression can vary across years and locations. Early work suggested that easily observable traits (e.g., seed color and branching) may be associated with resistance, although such indicators are not universally reliable (Gabr et al. 1998). Genetic studies under artificial inoculation and sick-plot conditions indicated that resistance can be controlled by recessive inheritance in some backgrounds (Thiyagu et al. 2007). However, evaluations of mutants, elite lines, and diverse germplasm repeatedly demonstrated substantial variability and occasional instability of reactions across seasons, reinforcing the need for robust, multi-year testing (Akhtar et al. 2011; Shabana et al. 2014; Bedawy and Moharm 2019; Farooq et al. 2019; Ghias et al. 2021). Recent effort has also focused the improvement pipeline through local pathogen characterization and complementary management approaches that may enhance resistance expression under stress-prone environments (Aydoğdu and Kurbetli 2024; Ahmed et al. 2024). Collectively, these studies provide the basis for identifying resistant sources and developing cultivars; the principal sesame varieties/lines reported as resistant or tolerant are summarized in Table 1.

### Overview of global sesame breeding programs

Sesame, an ancient oilseed crop, has evolved through both farmer selection and formal improvement programs, with early breeding focusing yield, oil content, and local adaptation. Over time, objectives expanded to include disease resistance to major diseases, drought and heat tolerance, improved seed quality (oil composition, lignans), and reduced harvest losses. In the twentieth century, research programs in Asia and Africa supported germplasm exchange and the establishment of national and international collections that underpin modern breeding techniques (Bedigian 2003). Sesame is also valued beyond oil production; seeds and leaves are used as food, and the leaves remain nutritionally important even after drying and storage (Bennett 1998).

In the last decade, sesame improvement has accelerated due to the integration of genomics and data resources into conventional breeding. Genome-enabled tools now support trait discovery and selection, including (i) reference genome initiatives and curated genomics platforms (e.g., SesamumGDB) that integrate genome assemblies and comparative resources across cultivated sesame and wild Sesamum species (Hengchun et al. 2024), (ii) pan-genome and multi-genome analyses that better capture structural variation and domestication signals relevant for breeding (Hengchun et al. 2024), and (iii) rapid mapping approaches such as high-density bin maps and QTL analyses for yield- and stress-related traits (Xu et al. 2024). Parallel advances include GWAS and multi-environment association studies targeting drought tolerance and oil/fatty acid composition, providing candidate genes and marker targets for breeding (Teklu et al. 2025; Kim et al. 2024; Elsafy et al. 2025). Improved use of genetic resources is also emerging through genomics-informed core/composite core collections derived from large genebank holdings, which can streamline parental selection and broaden the genetic base (Ruperao et al. 2025). In addition, proof-of-concept CRISPR/Cas editing has been demonstrated in sesame, indicating the feasibility of targeted trait improvement once priority genes are validated (You et al. 2022). Collectively, these developments are shifting sesame breeding from primarily phenotype-based selection toward genomics-assisted breeding, enabling faster development of varieties with improved yield stability, quality, and resilience under climate and disease pressure (Rauf et al. 2024; Sanni et al. 2024).

### Genetic diversity and conservation of sesame germplasm

**Fig. 3:**
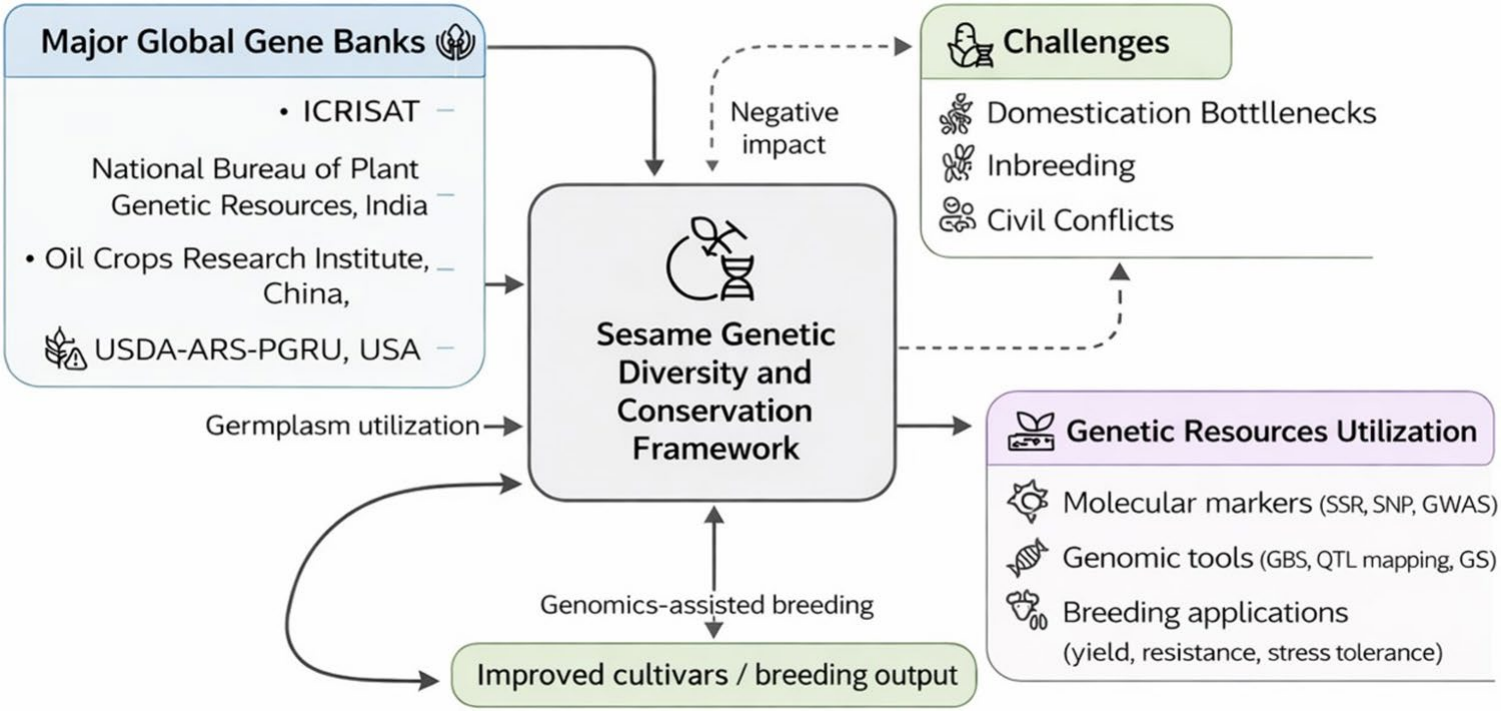
Conceptual framework of sesame genetic diversity and conservation. The diagram illustrates the role of major global gene banks (e.g., ICRISAT, NBPGR—India, Oil Crops Research Institute—China, USDA-ARS) in preserving sesame germplasm and supporting genetic diversity. These resources enable molecular marker studies and genomic tools that facilitate breeding applications, including yield improvement, stress tolerance, and disease resistance. Challenges such as domestication bottlenecks, inbreeding, and socio-political constraints can negatively affect genetic diversity and conservation efforts

Genetic diversity is essential for breeding; sesame is predominantly self-pollinating, and domestication and subsequent selection can reduce diversity in cultivated germplasm relative to wild relatives, underscoring the importance of broadening the breeding pool through landraces and wild Sesamum species (Wei et al. 2016; Teklu et al. 2022a, b). Wild relatives and landraces offer valuable traits like disease resistance, drought tolerance, and high oil content, but their potential remains underutilized. Molecular marker studies, such as RAPD, AFLP, and SNPs, reveal significant genetic diversity in wild species, aiding the expansion of the genetic base (Mesfer ALshamrani et al. 2022; Weldemichael and Gebremedhn 2023). Major sesame germplasm collections are conserved in national and international gene banks and are increasingly discoverable through public online catalogs (e.g., GRIN-Global and Genesys) (Fig. 3). For example, India’s National Genebank (ICAR-NBPGR) reports 10,520 sesame accessions in its seed genebank base collection (https://nbpgr.org.in/nbpgr2023/genebank-status-2/?utm/), and China’s National Mid-term Gene Bank hosted at the Oil Crops Research Institute (CAAS) maintains > 8,000 accessions, which has enabled the assembly of large diversity panels for genomics-assisted studies (Dossa et al. 2019). In the USA, the USDA-ARS sesame collection comprises 1,231 accessions, and recent work has screened the full collection for seed quality traits relevant to nutritional and oil quality improvement (Wang et al. 2025). While passport data and basic characterization are increasingly available, systematic multi-environment phenotyping remains uneven across collections; nevertheless, targeted evaluation is progressing (e.g., ICAR-NBPGR reports disease screening of 5,856 sesame accessions for phyllody and dry root rot) (Ruperao et al. 2024). Recent advances also include high-density genotyping and the development of core/composite core sets to accelerate utilization; for instance, ddRAD-seq-based analyses of thousands of accessions have been used to build representative subsets that capture broad allelic diversity for pre-breeding and association mapping (Ruperao et al. 2025). However, routine integration of genomics with standardized phenotyping and pre-breeding pipelines is still constrained by limited high-throughput phenotyping capacity and data harmonization in many programs (including in gene banks transitioning toward digital phenotyping approaches) (Kim et al. 2023). Breeding priorities in sesame continue to emphasize yield stability, oil quality, tolerance to abiotic and biotic stresses, and reduced capsule shattering/indehiscence (Islam et al. 2016). Finally, political instability and conflict remain contemporary risks to ex situ conservation; Sudan’s national genebank has recently reported losses and has pursued emergency safety duplication of seed samples in the Svalbard Global Seed Vault with international support (https://www.croptrust.org/news-events/news/sudans-national-genebank-begins-rebuilding-as-famine-threatens.com/). Ex situ conservation relies on major national repositories (e.g., ICAR-NBPGR, India; Oil Crops Research Institute/CAAS, China; RDA-Genebank, Republic of Korea; USDA-ARS, USA) and is increasingly supported by interoperable online databases (e.g., GRIN-Global and Genesys) (Teklu et al. 2021a, b; Yadav et al. 2022). Domestication-associated narrowing of the cultivated gene pool and external threats (including conflict) highlight the need for risk management and safety duplication (e.g., Svalbard Global Seed Vault). Molecular markers and next-generation sequencing support diversity assessment, core collection development, and genomics-assisted breeding targeting yield, oil quality, stress tolerance, and reduced capsule shattering (https://www.croptrust.org/what-we-do/programs/svalbard-global-seed-vault/?utm/). Pertinent websites and references offer supplementary information and access to their resources.

## Mechanisms of host resistance in sesame plants

### Host–Pathogen interactions dynamics

Plants have developed complex signaling mechanisms to respond to biotic and abiotic stresses (Bacete et al. 2018). Pattern-triggered immunity (PTI) and effector-triggered immunity (ETI) are key defense strategies in plant–pathogen interactions (Jones and Dangl 2006). PTI involves pathogen-associated molecular patterns triggering cell wall fortification and defense gene production, while ETI identifies effectors, leading to defense responses like reactive oxygen species (ROS) bursts and antioxidant enzyme activity (Kim et al. 2014). In sesame, *M. phaseolina* infects through roots or stems, causing vascular blockage and tissue necrosis. The pathogen releases hydrolytic enzymes to break down cell walls and generate ROS, inducing cell death (El-Fiki et al. 2004). In response, resistant sesame genotypes enhance cell wall fortification with lignin and suberin deposition (Radadiya et al. 2021). Resistance is further mediated by signaling molecules like salicylic acid, jasmonic acid, and ethylene. Resistant genotypes show better water-use efficiency, mitigating pathogen invasion, particularly under stress conditions. Understanding these interactions is crucial for breeding-resistant sesame cultivars. These signaling pathways form interconnected regulatory networks that ultimately control downstream physiological defenses, including lignification, ROS homeostasis, antioxidant enzyme activity, and antimicrobial metabolite production.

### Physiological and biochemical defense responses

**Fig. 4:**
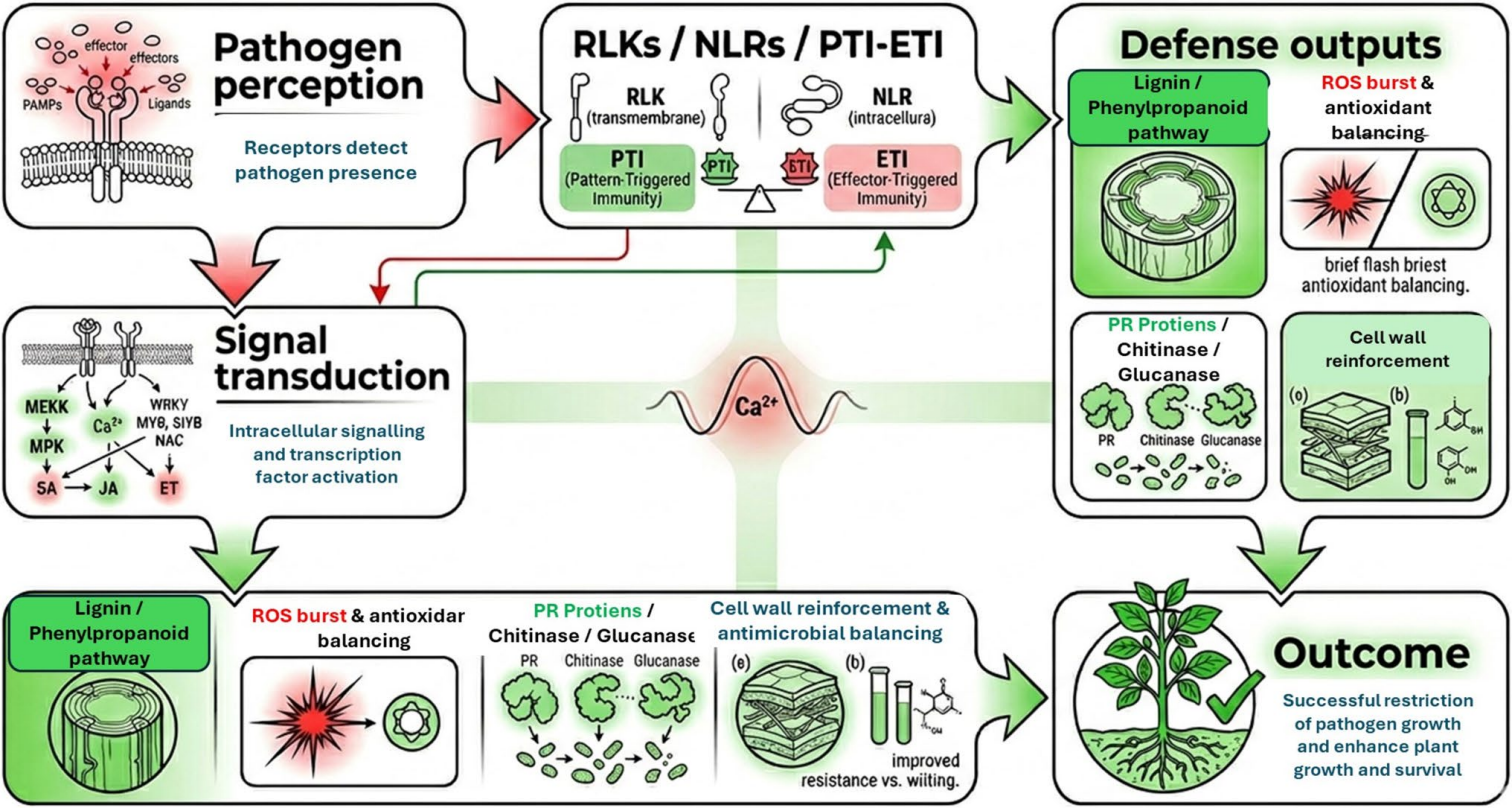
Integrated defense-response network associated with charcoal rot resistance in sesame. The diagram links pathogen perception and immune activation (PTI/ETI; receptor-like kinases and resistance proteins) with downstream signaling pathways (ROS, MAPK cascade, WRKY/MYB/NAC transcription factors, and SA/JA/ET signaling), which regulate key defense outputs including lignin biosynthesis, antioxidant enzyme activity, PR protein accumulation, and cell wall reinforcement. These coordinated responses limit fungal colonization and contribute to reduced disease severity and enhance plant survival.

Resistant sesame genotypes exhibit unique physiological and biochemical traits that enable them to withstand *M. phaseolina* infections (Fig. 4). These plants maintain efficient water and nutrient delivery, reducing wilting and early senescence. They compartmentalize the pathogen, preventing its spread. Biochemically, resistant genotypes show increased activity of defense-related enzymes such as peroxidases, polyphenol oxidases, and phenylalanine ammonia lyase (PAL), which help synthesize secondary metabolites like lignin, flavonoids, and phytoalexins that block pathogen growth (Yan et al. 2021). Lignin fortifies cell walls, while reactive oxygen species (ROS) activate defense genes. To maintain balance, resistant plants produce higher levels of antioxidant enzymes like superoxide dismutase (SOD) and catalase (CAT). Additionally, pathogenesis-related proteins, including β-1,3-glucanases and chitinases, help degrade fungal cell walls. Resistant genotypes also have higher levels of secondary metabolites such as tannins, alkaloids, and terpenoids, providing chemical defenses. Hormonal interactions among salicylic acid, jasmonic acid, and ethylene further strengthen their defense mechanisms (Dossa et al. 2017; Li et al. 2022; Yan et al. 2021, 2023; Ransingh et al. 2021). Recent evidence indicates that auxin signaling contributes to plant defense by regulating hormone crosstalk, transcriptional reprogramming, and adaptive growth responses, thereby influencing the balance between growth and defense under combined biotic and abiotic stress conditions (Ali et al. 2025a). Likewise, ROS should be viewed not only as damaging by-products but also as central signaling molecules whose production, scavenging, and redox-mediated crosstalk help coordinate defense activation, stress adaptation, and pathogen restriction (Ali et al. 2025b).

At the molecular level, these physiological and biochemical defenses are coordinated by interconnected regulatory networks rather than acting as isolated traits. Transcriptomic analyses of the sesame *M. phaseolina* interaction showed that resistant genotypes upregulate receptor-linked defense signaling, pathogenesis-related proteins, glutathione metabolism, secondary-metabolite biosynthesis, and multiple transcription-factor families, including WRKY, MYB, NAC, bZIP, and bHLH, together with phytohormone-responsive pathways (Radadiya et al. 2021; Yan et al. 2021). Recent analysis of sesame LRR-RLK genes under *M. phaseolina* stress further supports the role of membrane-localized perception and signal transduction in organizing downstream defense outputs (Yan et al. 2024). These signaling layers converge on the phenylpropanoid pathway and ROS regulatory systems, thereby linking induction of PAL-related lignin biosynthesis and cell wall reinforcement with antioxidant buffering, defense gene expression, and stress adaptation under infection (Shirai and Eulgem 2023). Taken together, available evidence indicates that charcoal rot resistance in sesame is governed by a coordinated gene regulatory network in which pathogen perception, hormone crosstalk, transcriptional regulation, and metabolic defense responses operate in an integrated manner. Genomic and statistical genetics approaches now enable rapid locus discovery for resistance and yield-related traits through QTL mapping, GWAS, and genomic prediction frameworks. Current evidence suggests that durable charcoal rot resistance in sesame depends on integrated defense perception, transcriptional regulation, and redox/cell wall-associated pathways, but robust loci stable across diverse germplasm and environments are still limited. Coupling these approaches with multi-omics and CRISPR-enabled functional validation will help accelerate durable resistance breeding outcomes.

## Breeding approaches for improving resistance to charcoal rot

### Conventional breeding and phenotypic screening strategies

Improvement of sesame varieties predominantly depends on traditional breeding techniques (Teklu et al. 2022a, b). Historically, the scarcity of genomic techniques and inadequate genetic databases for essential agronomic features hindered breeding initiatives (Dossa et al. 2016a). Conventional breeding has produced considerable genetic variety in sesame (Bisht et al. 1998; Zhang et al. 1999; Wei et al. 2015; Sehr et al. 2016). Characteristics including flowering and maturity time, plant height, branch count, capsule size, seed weight, and yield are widely used for variety characterization and breeding (Abdou et al. 2015; El Harfi et al. 2018; Teklu et al. 2021a, b).

However, robust characterization for improvement programs typically also includes the major yield components (capsules per plant, seeds per capsule, 1000-seed weight, biomass, and harvest index), capsule shattering/indehiscence, and lodging tolerance, because these traits directly determine yield stability and harvestability across environments (HobAllah 2003; Teklu et al. 2022a, b). In addition, seed quality traits such as oil content, fatty acid composition, and in some breeding contexts lignan-related quality attributes are important targets for cultivar development and market value. Stress adaptation traits (e.g., drought and heat tolerance indicators, early vigor, and root system attributes) are also increasingly prioritized to improve performance under rainfed and marginal conditions (Pandey et al. 2021; Jeyaraj and Beevy 2024).

Comprehending genetic diversity, heritability, and trait correlations are crucial for the usage of germplasm (Myint et al. 2020). Significant heritability and genetic progress facilitate efficient phenotypic selection. Research in India and Myanmar indicated significant heritability for characteristics such as plant height and capsule count (Divya et al. 2018; Aye and Htwe 2019). Correlations among traits enhance breeding efficiency. Sesame seed output exhibits a favorable correlation with plant height, branch count, and 1000-seed weight (Teklu et al. 2021a, b). Furthermore, oil content has an inverse connection with oleic acid, consistent with previous research (Dossa et al. 2016b; Teklu et al. 2022a, b; Singh et al. 2025). Assessing trait connections is essential for developing high-yield sesame cultivars with enhanced oil profiles. Screening for resistant varieties in sesame involves evaluating germplasm under natural and artificial disease pressure. Traditional field screening is influenced by environmental variability, while artificial inoculation methods provide controlled environments. Advanced phenotyping techniques, such as hyperspectral imaging and chlorophyll fluorescence measurements, improve accuracy but require specialized equipment. For charcoal rot specifically, standardized disease-response traits (incidence, severity scoring/AUDPC, plant survival, and yield loss under inoculated and hotspot conditions) should be integrated with agronomic and quality evaluation to enable consistent selection across environments and pathogen variability. Challenges remain, such as pathogen variability and lack of standardized protocols, necessitating the integration of conventional screening with molecular tools.

### Integrated disease management (IDM) as a supportive tool for resistance breeding

**Fig. 5:**
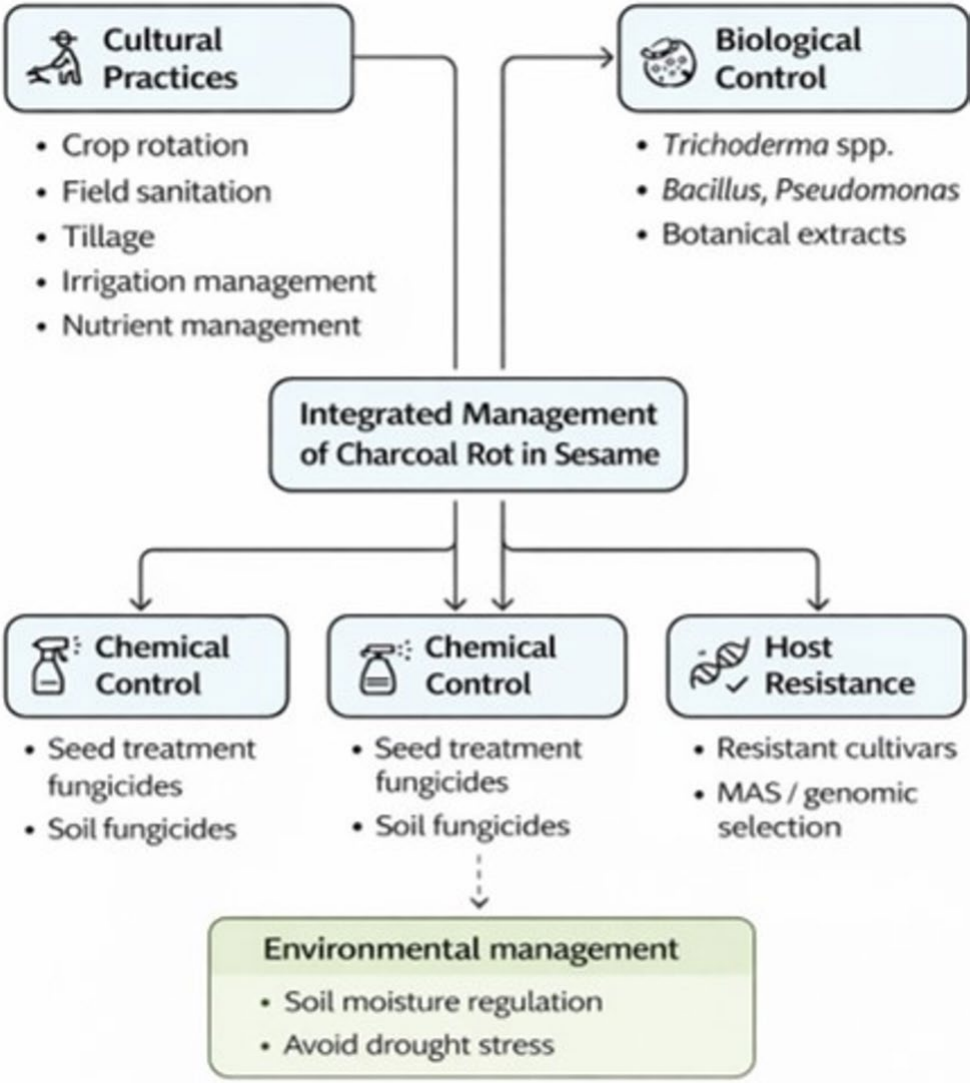
IDM strategies for controlling plant pathogens. Effective management begins with accurate pathogen diagnosis and then integrates cultural practices (crop rotation, residue management/sanitation, tillage, irrigation scheduling, and nutrient management), biological control agents (beneficial fungi, bacteria, and plant extracts), and host resistant through screening and breeding. The goal is to reduce inoculum, limit infection-favorable stress, and stabilize sesame seed yield under *M. phaseolina* pressure

Various approaches have been employed to control *M. phaseolina* both in the field and under laboratory conditions. Therefore, integrated disease management (IDM) is best viewed as a supportive strategy that complements resistance breeding, reduces disease pressure in farmers’ fields, and improves the reliability of phenotypic screening by limiting confounding environmental effects (Fig. 5). In sesame, effective IDM typically combines host resistance (when available), cultural and agronomic practices, biological control agents, and supportive inputs to protect seed yield and maintain seed quality (Mahalakshmi 2020).

Cultural practices remain the first line of defense because they reduce the inoculum reservoir and minimize stress conditions that predispose plants to charcoal rot. Crop rotation with non-host crop, sanitation (removal or management of infected residues), and appropriate tillage can lower *M. phaseolina* survival and reduce disease carryover (Vashisht et al. 2023). Disease risk is also strongly influenced by crop stress; therefore, irrigation scheduling, soil moisture conservation, and balanced nutrient management are critical components of IDM. Practices such as optimal plant spacing and timely harvest reduce plant stress and help avoid prolonged exposure to late-season infection pressure (Khaskheli et al. 2025). Soil health-oriented interventions (e.g., organic amendments and selected soil conditioners) may enhance microbial competition and suppress pathogen survival, while integrated nutrient management and weed control support overall plant vigor and reduce predisposition to infection.

Resistant or moderately resistant sesame varieties provide the most sustainable foundation for charcoal rot management, but performance can vary across environments and pathogen populations (Mahmoud et al. 2024). For this reason, resistance should be deployed together with agronomic best practices (stress avoidance and improved soil fertility) to stabilize resistance expression and protect sesame seed yield. Practical measures that support resistance-based control include the use of disease-free seed, appropriate crop establishment practices, and combining genetic screening/hybrid development with recommended crop management to reduce disease pressure and improve field performance (Pandey et al. 2023; Nanda et al. 2024).

Biological control is increasingly reported as an effective component of IDM for *M. phaseolina*, with benefits ranging from direct antagonism to induced resistance and competition in the rhizosphere. Plant extracts, beneficial fungi, beneficial bacteria, and emerging tools such as nanoparticles have all been evaluated for *M. phaseolina* suppression in sesame and other hosts (Karibasappa et al. 2020; Vashisht et al. 2023; Khamari et al. 2024; Mahato et al. 2024). Trichoderma species are among the most widely tested antagonists against *M. phaseolina. Trichoderma viride* Tv-1 reportedly reduced root and stem rot in sesame by 60.22% under in vitro and in vivo conditions (Rathore et al. 2020). Other reports also document strong suppression of charcoal rot by Trichoderma spp. in diverse hosts, supporting their broader utility as IDM components. Similarly, *T. harzianum* demonstrated high efficacy against charcoal rot in green and black grams (in vitro: 79.63%, in vivo: 86.67% (Iqbal et al. 2020)) and in soybean with 81.85% inhibition (in vivo; Rahman et al. 2021). *T. hamatum* inhibited 51.55% of root and stem rot in peanuts (Martínez-Salgado et al. 2021), while *T. harzianum* and *T. viride* suppressed charcoal rot in cowpea by 73.33% and 65.59%, respectively (in vitro; Nitika Kumari et al. 2022). Other studies include *T. asperellum* (60.1%) in chia plants (in vivo; Mergawy et al. 2022), *T. longibrachiatum* (75.98%) in cotton (Degani et al. 2023), and *T. harzianum* (78.9%) in sesame (in vitro) (Nanda et al. 2024).

Among bacterial biocontrol agents, *Pseudomonas fluorescens* and *Bacillus subtilis* are frequently reported as effective against MP. In sesame, *P. fluorescens* Pf-1 inhibited root and stem rot by 48.86% (Rathore Jainpal et al. 2020), and another study reported 71.38% inhibition of root rot by *P. fluorescens* (Karibasappa et al. 2020). *B. subtilis* has also been reported as highly suppressive in sesame under in vitro and in vivo conditions (Abd et al. 2020), while additional studies report moderate inhibition against charcoal rot in sesame and strong activity in other hosts (Nanda et al. 2024; Degani et al. 2024). Collectively, these findings support the inclusion of microbial antagonists in IDM packages, particularly where resistant cultivars are not yet consistently available.

Plant extracts like neem, garlic, and ginger are frequently cited for antifungal activity and may be used as supportive options where validated locally. Yeasts have also been explored for competitive exclusion and antagonistic effects. In addition, nanoparticles (e.g., silver- or copper-based materials) have been reported to exhibit antifungal activity and may be integrated cautiously into IDM where environmental and safety considerations are addressed (Moradi et al. 2024).

### Advances in modern breeding technologies

**Fig. 6:**
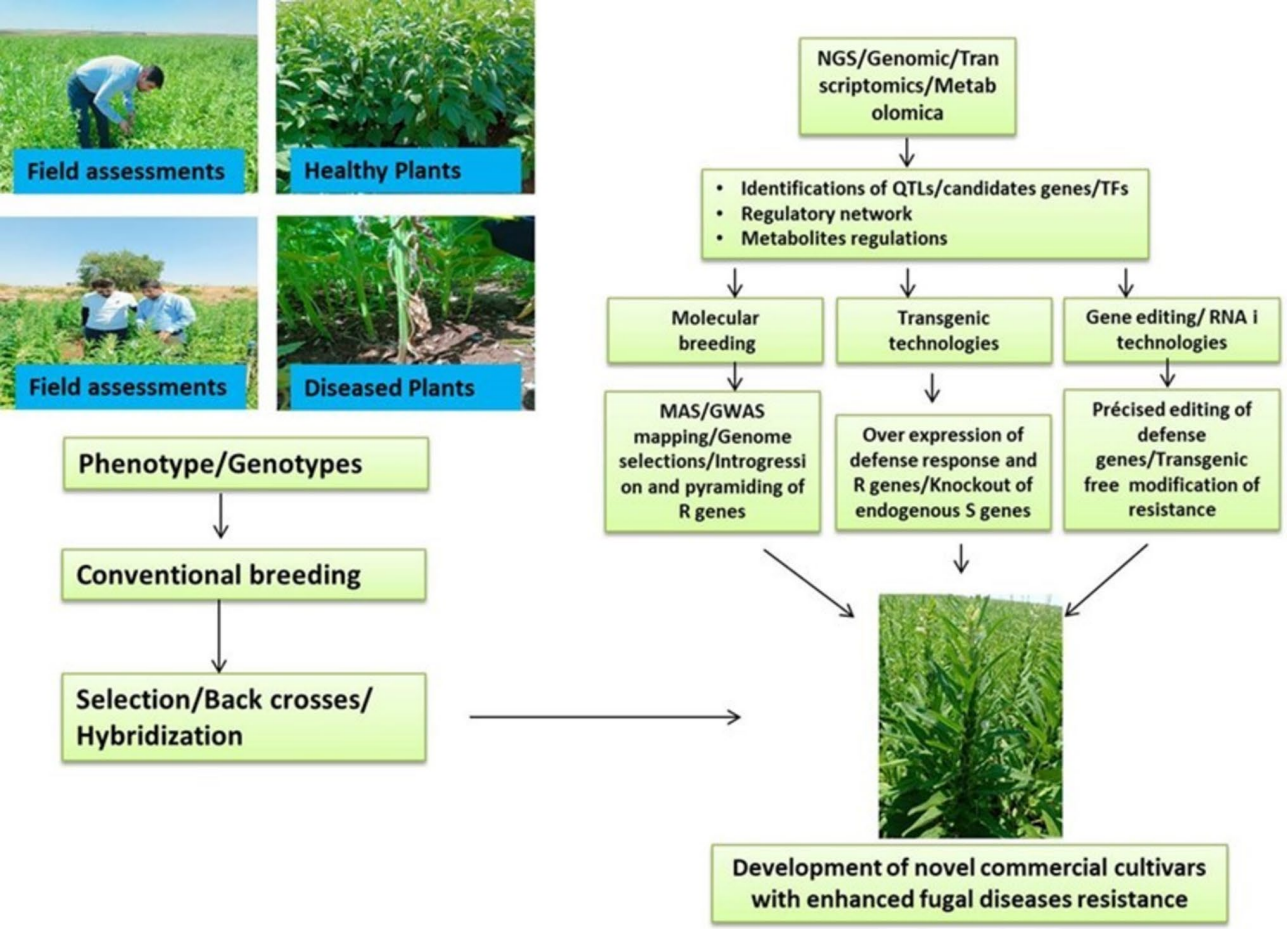
Phenotyping to genotypic, a step-by-step presentation of the development of novel commercial sesame cultivars

Modern breeding techniques leverage genomic and biotechnological tools to accelerate the development of resistant sesame varieties (Li et al. 2023; Seay et al. 2024). By targeting specific resistance genes or genomic regions, these approaches enable precise improvements and reduce the breeding cycle. Techniques like marker-assisted selection (MAS), genomic selection (GS), and genome-wide association studies (GWAS) have transformed breeding programs, while gene-editing technologies, such as CRISPR-Cas9, offer unprecedented precision in trait manipulation (Fig. 6).

## Genomic-Enabled Breeding Strategies

### Sesame genome resources and molecular marker development

Whole-genome sequencing is a vital tool for understanding the genetic basis of complex traits, enabling targeted genomic strategies for crop improvement. Resequencing reveals extensive genetic variation including SNPs, CNVs, and InDels facilitating precise trait characterization (Kumar et al. 2024). These markers are instrumental in identifying genes associated with resistance to soil-borne fungal pathogens, supporting varietal development and sustainable agriculture (Nadeem et al. 2018). Sesame genome size ranges from 369 to 375 Mb, depending on genotype and sequencing approach (Sabag et al. 2021). The Sinbase 1.0 platform (http://orci-genomics.org/Sinbase/) serves as a key genomic repository, hosting data on over 401,063 genes, 406 markers, and 16,296 scaffolds. The mitochondrial genome comprises 22 circular chromosomes totaling 724,998 bp, with 436 mitochondrial genes. Additionally, more than 187 SSRs, 7,000 validated, and over 100,000 unvalidated SSR markers have been reported (Wang et al. 2023).

Various markers including AFLP, RAPD, ISSR, EST-SSR, cDNA-SSR, SNPs, InDels, and gSSRs have been applied in sesame genetic studies (Singh and Kumar, 2022). Advanced techniques like GCIM, ICIM, CIM, SLAF-seq, and genotyping by sequencing (GBS), especially useful for species with low polymorphism, have enabled the development of over 18 genetic linkage maps (Li et al. 2021). The first map, created in 2009 with 284 PCR markers, was followed by a high-density SLAF-seq map in 2013 (Xu et al. 2024). Later advancements include ultra-dense maps with over 30,194 SNPs (Wei et al. 2016) and the application of RAD-Seq (Wu et al. 2014), culminating in recent ultra-dense maps (Rauf et al. 2024). These developments have significantly enhanced marker-assisted selection (MAS), accelerating the genetic improvement of key traits such as pathogen resistance, and advancing sesame’s role in food security and sustainable agriculture. More recent resource developments further strengthened molecular marker discovery in sesame. Updated genomics platforms now integrate cultivated and wild *Sesamum* genomes and comparative tools for marker mining and candidate gene prioritization (Hengchun et al. 2024), while new high-density genetic maps and QTL analyses continue to refine marker intervals for defense- and yield-related traits (Xu et al. 2024; Teklu et al. 2025). At the gene family level, recent expression-guided analyses of receptor-like kinase pathways under *Macrophomina* stress have also expanded the pool of defense-associated markers for downstream validation and breeding use (Yan et al. 2024).

### Marker-Assisted Selection (MAS) for charcoal rot resistance

MAS uses molecular markers linked to resistance traits to enhance genotype selection in sesame breeding. QTL mapping and genomic studies have identified markers for charcoal rot resistance, aiding in trait identification and molecular breeding (Khan et al. 2020). The sesame genome is 554.05 Mbp, comprising a core genome of 258.79 Mbp and a dispensable genome of 295.26 Mbp, containing 26,472 orthologous gene clusters, including 15,890 variety-specific genes (Yu et al. 2019). These genomic resources are vital for sesame improvement. MAS allows breeders to evaluate large populations for resistant traits efficiently, reducing reliance on phenotypic screening and conserving resources (Cobb et al. 2019). Molecular markers related to lignin production, cell wall integrity, and stress-related enzymes are key for charcoal rot resistance. MAS combined with backcrossing has proven effective in consolidating resistance genes, improving the durability of resistance (Sharma et al. 2023). Resistance to *M. phaseolina* in sesame involves several minor genes and QTLs, including genes for PR proteins, lignin production, and transcription factors (Mahmoud et al. 2024). Wang et al. (2017) mapped 14 QTLs for charcoal rot resistance in sesame and reported that key QTL intervals contain candidate defense-related genes, including a cluster of receptor-like kinase (RLK) genes in the major-effect QTL region and NBS-encoding resistance genes in the regions for the stable QTLs (qCRR8.2 and qCRR8.3). Transcriptomic analyses show overexpression of defense genes in resistant genotypes. SNPs identified through GWAS are associated with lignin production, ROS scavenging, and phytohormone signaling (Li et al. 2023; Shirai and Eulgem 2023). Gene editing via CRISPR-Cas9 holds promise for improving resistance (Rauf et al. 2025). Despite these advances, further research is needed to identify and validate additional resistance genes and QTLs for better productivity and stress resilience.

### Genome-Wide Association Studies (GWAS) and Genomic Selection (GS)

Genome-wide association studies (GWAS) and genomic selection (GS; genome-wide prediction) are increasingly used to dissect complex traits and accelerate genetic gain in crop improvement. In sesame, GWAS enabled by GBS and high-density SNP resources has already identified robust loci for several seed quality and agronomic traits, including vitamin E (tocopherol) composition (He et al. 2019), seed coat color (Cui et al. 2021), and oil content and fatty acid composition evaluated across environments (Elsafy et al. 2025), as well as protein and oil content in multi-environment panels (Kefale et al. 2025). Likewise, recent work has demonstrated the feasibility of genomic prediction for multiple agronomic traits in sesame, with improved accuracy when multi-environment data are incorporated an essential first step toward operational GS in breeding programs (Sabag et al. 2023).

**Fig. 7:**
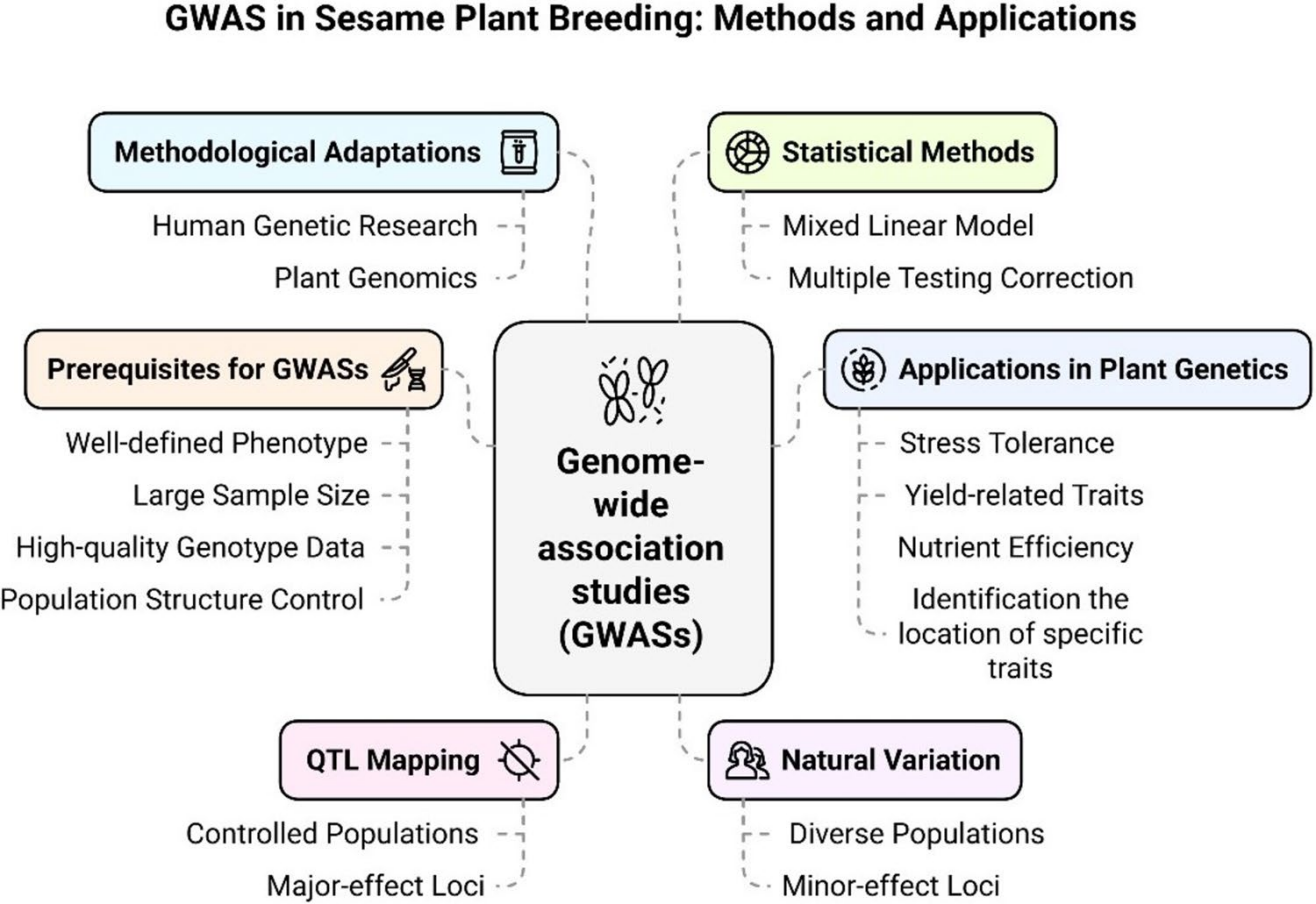
GWAS in sesame plant breeding, their methods, and application

In contrast, charcoal rot resistance in sesame has so far been investigated mainly through biparental linkage/QTL mapping and transcriptome-based candidate gene discovery, rather than large-scale GWAS panels. For example, QTL mapping identified multiple loci for charcoal rot resistance across field environments and proposed candidate disease-response genes (Wang et al. 2017), while transcriptome comparisons between resistant and susceptible genotypes highlighted defense-related signaling and receptor-like kinases involved in the response to M. phaseolina (Yan et al. 2021). Therefore, there is an urgent need to develop well-characterized association panels phenotyped under standardized charcoal rot screening (field and controlled inoculation), coupled with dense SNP genotyping and curated genomic resources, to enable GWAS discovery and downstream GS implementation for charcoal rot resistance (Fig. 7). Wei et al. (2016) identified molecular mechanisms of resistance to charcoal rot in sesame, revealing differentially expressed genes (DEGs) between resistant and susceptible varieties. Additionally, Tesfaye et al. (2022) found 21 significant marker–trait associations (MTAs) for yield-related traits, aiding in the development of high-yielding varieties. Foundational resources such as integrated sesame functional genomics databases and large SNP datasets further support this direction (Wei et al. 2017). Recent studies from 2024 to 2025 also indicate that the pipeline from resistance phenotyping to marker deployment is becoming more practical in sesame. Breeding studies using resistant and susceptible parents have refined useful donor backgrounds for charcoal rot improvement (Mahmoud et al. 2024), and candidate gene validation under artificial inoculation has identified defense-related targets that can be connected to marker development and functional assays (Najar and Gangophadhay 2024). In parallel, contemporary GWAS and germplasm-mining studies are expanding the set of high-confidence SNPs and useful accessions available for trait introgression and genomic selection, even when the focal traits are broader than charcoal rot itself (Kefale et al. 2025; Wang et al. 2025). Together, these recent advances substantially improve the timeliness of the genomic breeding discussion and reinforce the need to combine updated marker resources with rigorous disease phenotyping.

### CRISPR-Cas system, Base Editing, and Prime Editing for Targeted Resistance Improvement

Genome-editing technologies including CRISPR-associated nucleases (Cas9/Cas12a), base editors, and prime editors provide accurate and powerful, sequence-specific tools to modify gene linked to resistance against *M. phaseolina*. Conventional CRISPR nucleases induce targeted double-strand breaks that are repaired primarily by non-homologous and joining enabling gene knockouts or allele disruption, and they can be multiplexed to manipulate quantitative/polygenic resistance networks in sesame (You et al. 2022). Genome editing has also been used to engineer disease resistance in other crops via disruption of susceptibility genes or cis-regulatory elements (Nekrasov et al. 2017; Oliva et al. 2019). Accordingly, candidate targets for charcoal rot improvement include negative regulators of immunity and genes involved in phenylpropanoid/lignin biosynthesis, pathogen perception, cell wall fortification, reactive oxygen species (ROS) homeostasis, and hormone signaling pathways (salicylic acid and jasmonic acid) that coordinate defense responses (Thomazella et al. 2021; Rauf et al. 2024). Despite its potential, gene editing in sesame encounters technical and regulatory obstacles. Effective transformation methods and the identification of target genes are essential for successful deployment. Furthermore, such edits can, in principle, increase resistance without stable integration of exogenous DNA when delivered as transient DNA, RNA, or ribonucleoproteins.

Beyond nuclease editing, precision “writing” tools may be particularly useful for engineering favorable alleles in sesame. Base editing uses a Cas nickase fused to a deaminase to introduce predictable single-nucleotide substitutions (typically C → T or A → G) without creating double-strand breaks or requiring a donor template (Komor et al. 2016; Hua et al. 2019; Ren et al. 2021). This approach can recreate natural resistance-associated polymorphisms or introduce stop codons in susceptibility genes; for example, base editing of eIF4E to mimic a natural polymorphism conferred potyvirus resistance in Arabidopsis (Bastet et al. 2019). Prime editing further expands edit types by coupling a Cas9 nickase to a reverse transcriptase and a prime-editing guide RNA, enabling targeted small insertions/deletions and all base substitutions again without double-strand breaks or donor DNA (Anzalone et al. 2019). Prime editing has been demonstrated in crops such as rice and wheat and is being optimized for higher efficiencies (Lin et al. 2020; Tang et al. 2020; Tian et al. 2025). In sesame, base/prime editing could accelerate translation of GWAS/QTL signals into elite cultivars by precisely rewriting causal SNPs or regulatory motifs, while minimizing linkage drag from conventional introgression. Despite this promise, gene editing in sesame faces technical and regulatory constraints. Efficient, genotype-flexible transformation and regeneration systems, reliable target validation, and rigorous assessment of on-/off-target outcomes remain prerequisites for deployment. Regulatory treatment of gene-edited crops also varies among countries, influencing testing and commercialization pathways (Buchholzer and Frommer 2023). Nevertheless, integrating CRISPR nucleases with base and prime editing together with high-resolution mapping, functional genomics, and breeding pipelines provides substantial potential to develop sesame cultivars with improved resistance to charcoal rot (Vashisht et al. 2023).

## Future directions and opportunities in resistance breeding

### Broadening the resistance gene pool: wild relatives, landraces, and pre-breeding

Future work should move from general calls for wider diversity toward targeted use of germplasm that is most likely to contribute durable resistance. Priority should be given to wild Sesamum species landraces from drought- and heat-prone regions, because these environments are also favorable for charcoal rot development. Developing structured pre-breeding populations, bridging crosses, and resistant donor lines from such materials will be essential to transfer useful alleles into elite backgrounds while minimizing linkage drag.

### Pangenome-informed discovery and deployment of resistance

Reference genome-based analyses alone may miss resistance-relevant variation. Future studies should therefore integrate pangenome resources to capture structural variants and presence/absence variation associated with defense traits. Combining pangenomics with GWAS, haplotype analysis, and multi-environment validation will help identify robust resistance loci and support the development of breeder-friendly diagnostic markers for deployment in marker-assisted and genomic selection pipelines.

### High-throughput phenotyping, AI, and precision agriculture

Progress in resistance breeding will also depend on more standardized and scalable phenotyping. UAV-based imaging, thermal sensing, hyperspectral tools, and AI-assisted image analysis can improve early stress detection and more objective disease scoring under field conditions. When integrated with soil moisture sensing and climate-based disease forecasting, these precision agriculture tools can help identify disease hotspots, improve screening accuracy, and support environment-specific selection strategies.

### Functional validation and systems-level understanding

A major gap remains between candidate gene discovery and confirmed biological function. Future research should prioritize functional validation of the most promising resistance-associated genes and regulatory hubs identified through transcriptomics and other omics approaches. Multi-omics integration will be especially valuable for clarifying how drought stress modifies defense signaling, lignification, ROS regulation, and other pathways relevant to charcoal rot resistance.

### Global collaboration, data sharing, and translational pipelines

**Fig. 8:**
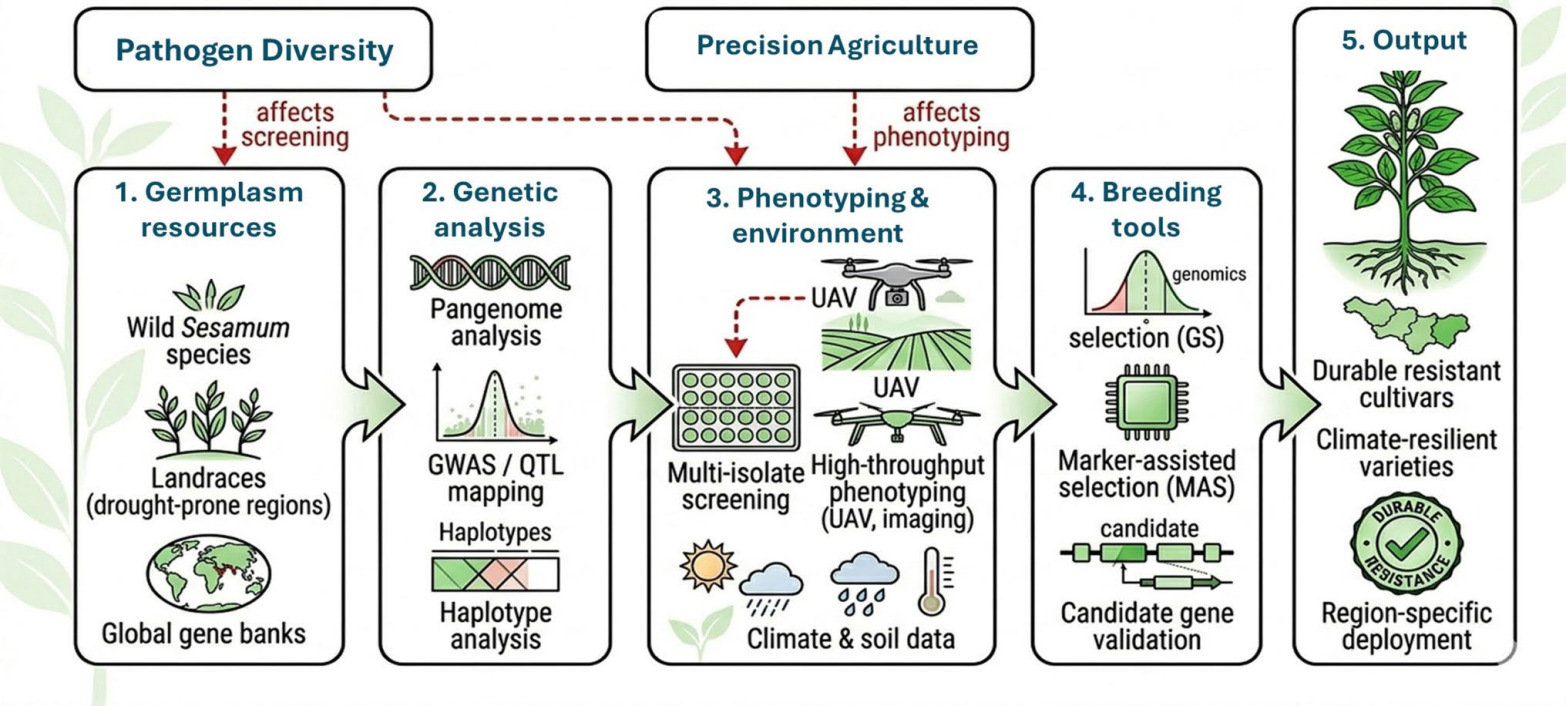
Strategic framework for sesame resistance breeding against charcoal rot. The diagram summarizes an integrated pipeline from germplasm resources and genomic analyses to phenotyping and genomics-assisted breeding for the development of durable, climate-resilient sesame cultivars

Global collaboration in sesame research including initiatives such as the International Sesame Genome Consortium will be essential to accelerate resistance breeding by enabling shared germplasm exchange, coordinated multi-location trials, standardized phenotyping pipelines, and interoperable genomic datasets (Fig. 8). Harmonizing regulatory and biosafety discussions (especially for gene-edited products) and investing in shared infrastructure and training can reduce duplication and speed delivery of improved cultivars. Ultimately, these coordinated efforts support the development and dissemination of resilient sesame varieties that contribute to food and nutritional security.

## Conclusions

Charcoal rot caused by *M. phaseolina* is a major threat to sesame production, particularly in drought-prone regions where disease severity and yield losses are often amplified. This review highlights the evidence that (i) the pathogen's broad host range and stress-associated aggressiveness complicate management, (ii) sesame resistance is predominantly polygenic/quantitative, making phenotypic selection alone slow and environmentally confounded, and (iii) progress has been limited by narrow cultivated diversity, variable screening methods, and incomplete functional understanding of resistance pathways. Recent advances in genomic resources and molecular breeding provide a practical pathway to accelerate resistance improvement. Current evidence suggests that durable charcoal rot resistance in sesame depends on integrated defense perception, transcriptional regulation, and redox/cell wall-associated pathways, but robust loci stable across diverse germplasm and environments are still limited. Specifically, integrating robust phenotyping with GWAS/QTL discovery, validated molecular markers, and modern breeding strategies (marker-assisted selection and genomic selection) can increase selection accuracy for quantitative resistance. In parallel, high-throughput phenotyping and AI-based analytics can scale screening and reduce subjectivity, while functional genomics and gene-editing approaches can help validate and manipulate key defense regulators where transformation and regulatory contexts allow.

Significance and application in breeding programs: The combined framework outlined here is directly actionable for breeding pipelines: It enables (1) systematic discovery of resistant donors from landraces/wild materials, (2) conversion of loci into deployable markers for rapid introgression and pyramiding, (3) improvement of selection under drought-linked disease pressure through standardized, multi-environment testing, and (4) prioritization of validated candidate genes and pathways as targets for precision improvement. Together, these steps can shorten breeding cycles, increase the probability of durable resistance, and support stable sesame yields under climate variability.

Limitations and research gaps: Because this work is a synthesis of available studies, conclusions are constrained by limitations in the underlying literature, most notably variability in inoculation/phenotyping protocols, uneven multi-location validation, limited functional validation of candidate genes in sesame, and incomplete characterization of pathogen diversity and genotype × environment interactions (especially drought). A major unresolved question is the precise molecular basis of the sesame *M. phaseolina* interaction under combined heat and drought stress, particularly how stress signaling, hormone crosstalk, ROS homeostasis, and defense gene activation are coordinated to determine susceptibility or durable resistance. Addressing this gap will require integrated transcriptomic, physiological, and multi-environment validation studies using both contrasting host genotypes and diverse pathogen isolates.
